# Ensuring Rich Rigor of Qualitative Methodologies in Behavior Analytic Research

**DOI:** 10.1007/s40617-025-01150-0

**Published:** 2026-02-04

**Authors:** Daria K. Lorio-Barsten, Selena J. Layden

**Affiliations:** 1 William & Mary, 301 Monticello Ave., Williamsburg, VA 23187 USA; 2 Old Dominion University, Norfolk, VA USA

**Keywords:** Qualitative, Methodology, Research Rigor

## Abstract

Quantitative methods remain the hallmark of research in applied behavior analysis. Yet, such methods frequently fail to capture the nuances of context where behavior analysis is practiced. Therefore, qualitative methods can provide complementary means to gain deeper insight into changes in socially significant behavior. We believe that researchers within the field of behavior analysis have much to gain from embracing qualitative methodologies. We propose that more researchers can and should consider conducting rigorous qualitative research to elevate the voices of the participants and relate the depth and complexities of their nuanced experiences. This article discusses Tracy’s “big tent” quality criteria to offer guidance to qualitative researchers on ensuring their studies possess high quality. Two recent qualitative research studies, conducted by the authors, are offered as illustrations to demonstrate how “big tent” criteria can be applied to design and conduct meaningful and rigorous research of high quality. We hope that these examples can encourage other researchers in the field of behavior analysis to feel more confident and see value in conducting qualitative research to gain a more comprehensive understanding of human behavior.

As behavior analysis emerged as a recognized science, researchers developed new methods that did not rely on large samples or statistical analyses, leading to publishing challenges because their work conflicted with the prevailing research standards of the time (Cooper et al., 2020). The questions that ignited behavior analysts’ curiosities were more readily answered using the now well-respected methodology in behavior analysis, single-case design (SCD; Baer et al., 1968; Ledford & Gast, 2018)). This is a quantitative approach where “participants serve as their own control” (Ledford & Gast, 2024, p. 11). SCD is useful for behavior analysts, who often focus on studying behavior change within a small sample or even one individual. Commonly, behavior analysts investigate the effects of an intervention and observe the change in behavior that may or may not occur. Although SCD is effective for determining whether an intervention leads to behavior change, this method alone is insufficient to fully understand the full complexity of human behavior.

By expanding the application of behavior analysis into various settings, we have begun to recognize the importance of variety in contexts. Although the importance of environment has been recognized since the inception of our field, context offers greater nuance. For example, school systems differ greatly from clinical settings and bring setting-specific situational variables (e.g., other students’ behaviors, learning context). Quantitative methods can frequently fail to capture such nuances (Silverio et al., 2022). Therefore, qualitative methods can provide complementary means to gain deeper insight into changes in socially significant behavior (Corbin & Strauss, 2015), which is included in the definition of applied behavior analysis (ABA; Cooper et al., 2020). Quantitative and qualitative methods are not dueling research approaches. Rather they share the same general goal: to “understand the world better” (Aspers & Corte, 2019). However, quantitative methods remain the hallmark of research in ABA. Recently, qualitative research within ABA has experienced noted growth, though most qualitative methods used appear to focus on addressing the social validity of interventions (e.g., Leko, 2014; Nicolson et al., 2020). Burney et al. (2023) proposed qualitative methodologies can expand research not only in the areas of social validity but also in more diverse topics (e.g., cultural and political movements, compelling societal issues, climate change research). We welcome this shift and echo the idea that qualitative research can bring additional value to the field through a broader application than is currently used.

## Utility of Qualitative Research in Behavior Analysis

Qualitative research focuses on behavior and its subjective meanings (Creswell, 2009). It explores individuals’ own accounts of their behaviors, attitudes, and motivations, providing rich data (Creswell, 2009). We compare these accounts to verbal behavior and the idea of private events in behavior analysis. Individuals’ meanings of their lived experiences can offer additional insights into the complex experiences and contexts that behavior analysts encounter.

Whereas quantitative researchers strive to approach an inquiry from an objective stance, often taking on the role of an outside observer, and seeking to minimize subjective influences in data collection and analysis, qualitative researchers’ approach differs (Mohanasundari et al., 2023). Instead, they embrace the researcher’s role as an instrument, in which the researcher’s knowledge, expertise, and perceptions factor into data collection, analysis, and interpretation (Pezalla et al., 2012). Although this approach can appear to be more subjective in comparison, it offers a deeper, more nuanced, and contextually situated understanding of an inquiry (Mohanasundari et al., 2023). These differences in approaches lead to methodological differences as well: quantitative researchers use numerical data to test a hypothesis, qualitative researchers focus on non-numerical data to gain rich insights from the study’s participants (Mohanasundari et al., 2023). Rather than manipulate an independent variable to determine its impact on a dependent variable, qualitative researchers focus on “observation of naturally occurring events” and make meaning of them (Ledford & Gast, 2024, p. 9). Although methodological techniques within qualitative research vary, each seeks to provide a deep understanding of the research inquiry (Ledford & Gast, 2024).

When beginning any research study, it is important to determine the research question. Different questions are better suited to be answered by different methodologies. For example, questions about characteristics of population could be answered by quantitative methods such as survey methodology or group designs. Questions about the effectiveness of an intervention for a particular individual lend themselves to SCD. Additionally, many other questions with frequency-based underpinnings, even regarding private events, could be addressed through a quantitative methodology. For example, a behavior therapist could count the number of their own negative thoughts that occur about their workplace. Yet, other types of questions which dive deeper into human experience are difficult to answer by solely using quantitative investigation. For example, questions about private events, behavior that has already occurred or lived experiences, may begin to be answered by quantitative studies, but may not reach the desired depth of the phenomenon under study, lend themselves well to qualitative research (Tenny et al., 2017). In an earlier example, a behavior therapist who is counting their negative thoughts about their workplace would not gain an understanding of why the thoughts are occurring from only using a frequency count. Additionally, qualitative research can also complement quantitative methodologies by exploring new areas of research and discovering relevant variables for further study (Corbin & Strauss, 2015).

Despite its potential utility, a lack of understanding of qualitative research designs can lead to undue criticism of these methods and overall misconceptions that qualitative research lacks rigor and consists of researchers’ opinions (Morgan, 2024). Unlike quantitative researchers, who have well-established quality criteria for validity, reliability, generalizability, and objectivity (e.g., Cook et al., 2014), qualitative researchers face a “methodological landscape [which] possesses a large variety of concepts and discussions around quality” (Tracy & Hinrichs, 2017, p. 1) and lack a consensus regarding standards needed to demonstrate rigor (Noble & Smith, 2015). Some qualitative researchers have even argued against establishing a universal set of quality criteria, as they worry that a checklist may not be sufficiently flexible to accommodate the wide variety of approaches in qualitative studies (Johnson et al., 2020) or result in a methodological incongruence (Braun & Clarke, 2024). It is important to note that qualitative researchers do find common ground in valuing rigor and trustworthiness (Braun & Clarke, 2024). Their differences lie not in conducting rigorous research, but rather a shared operational definition of rigor based on their varied methodologies and theoretical frameworks. However, a common language around best practices offers an option for qualitative researchers to systematically structure their work regardless of their preferred theoretical frameworks or methodologies (Braun & Clarke, 2024; Tracy & Hinrichs, 2017).

Although several frameworks for qualitative quality criteria exist and may be appropriate to use, Tracy (2010) offers a model of a simple structure that has utility for all qualitative methodologies and is accessible to novice qualitative researchers. The eight criteria include: a worthy topic, rich rigor, sincerity, credibility, resonance, significant contribution, ethics, and meaningful coherence.

## “Big Tent” Criteria

We chose to focus on Tracy’s (2010) framework because it can be universally applied to different qualitative methodologies and introduces a level of rigor that, while not exactly the same as what is seen in quantitative studies, does demonstrate overlap in concepts. Furthermore, we interpret these quality criteria as closely aligned with the dimensions of applied behavior analysis (Baer et al., 1968), which enhances their accessibility for applied behavior researchers.

### Worthy Topic

The first criterion of quality research is a worthy topic, which refers to a researcher’s selection of a focus that is “relevant, timely, significant, interesting, or evocative” (Tracy, 2010, p. 840). Similar to the *applied* dimension of ABA described by Baer et al. (1968), which focuses on examining socially significant behavior, Tracy’s worthy topic criterion was designed to identify a research topic worth investigating. Tracy (2010) offered several examples to describe the topic’s worth. Worthy topics can align with disciplinary priorities and offer theoretical or conceptual value to broaden the field of study. They also can be topics that question previously held assumptions and challenge ideas. Although Tracy’s explanation of a worthy topic is broad, the overarching idea is to encourage a researcher to select a meaningful topic that interests or is significant to others.

### Rich Rigor

The second criterion of high-quality research, as identified by Tracy (2010), is rich rigor, which is essential for increasing the quality of research. Although it is not necessarily seeking a functional relationship, the *analytic* dimension of behavior analysis (Baer et al., 1968) is similar in that both aim for quality research. Rich rigor combines the requisite variety, face validity, and adequacy of the research process. Requisite variety, a term borrowed from cybernetics, refers to the need for the tool to be at least as complex as the phenomenon under study (Weick, 2007). In qualitative research, the researcher serves as the instrument (Pezalla et al., 2012), using their worldview, knowledge, assumptions, and approach to research as part of the process (Corbin & Strauss, 2015). Tracy (2010) explains that when researchers have extensive knowledge and have generated abundant data, they can see nuance and complexity, thus becoming finely tuned instruments. However, requisite variety alone is insufficient for rich rigor. A researcher must also demonstrate face validity and adequacy of the research process. These are familiar concepts to quantitative researchers as well. Face validity speaks to the plausibility of the research; does the study measure or reflect what it states it is studying? Additionally, the adequacy of the research is needed for rich rigor. Such adequacy is demonstrated when a reader can follow the researcher’s logic throughout all aspects of the study and judge the process to be appropriate (Strauss & Corbin, 1998).

### Sincerity

The third criterion of quality research is sincerity. It addresses the researcher’s earnestness throughout the study. Tracy (2010) described sincerity as a researcher’s “honesty and authenticity with one’s self, one’s research, and one’s audience” (p. 842). Sincerity combines self-reflexivity and transparency. Reflexivity is the researcher’s ongoing reflection of their values, beliefs, experiences, and assumptions during the study (Corbin & Strauss, 2015), paying specific attention to how they shape the research process (Hall & Callery, 2001). In comparison, transparency denotes the researcher’s honesty about the research process. While qualitative research is not necessarily focused on replicability due to its focus on specific individuals at specific moments in time, the dimension of *technological* is related in that procedures should be clearly described (Baer et al., 1968).

### Credibility

Related, credibility is the fourth criterion, which addresses the trustworthiness of the study’s findings. Tracy (2010) detailed how thick description, triangulation, multivocality, and member reflections contribute to the study’s quality. Thick description requires sufficient detail of the phenomenon to show, rather than only tell, the findings in a way that transports the reader into the research setting (Creswell & Creswell, 2018; Tracy, 2010). Triangulation includes methods, data type, theory, and researcher triangulation (Morgan, 2024). Data source or methods triangulation refers to using a variety of origins of data (e.g., people and documents), and data type triangulation is the incorporation of multiple methods (e.g., interviews, observations) to generate data (Morgan, 2024; Patton, 2015). Multivocality is when researchers draw on varied voices in reporting the analyzed data, using thick descriptions to represent a diverse range of perspectives within a study (Tracy, 2010). Member reflections ensure the researcher’s accurate understanding of the participants’ shared perspectives and experiences (Creswell & Creswell, 2018) and bring participants into the dialogue with a researcher to question, critique, and offer feedback on the study’s findings (Tracy, 2010). Combined, these components of thick description, triangulation, multivocality, and member reflections help improve credibility of the study’s results, thus increasing the overall quality of the study.

### Resonance

The fifth criterion of quality research is resonance, a study’s ability to promote general regard for the topic and affect the readers. Tracy (2010) identifies aesthetic merit and generalizability as aspects of resonance that contribute to the quality of research. Aesthetic merit refers to the researcher reporting the results in a manner that tells a story, draws the reader in, and engages them, offering generalizability. While *generality* is a dimension of behavior analysis (Baer et al., 1968), it refers to the findings of the study generalizing to different people or settings, for example. However, in qualitative research, generalizability means a potential for readers to transfer the study’s results to their own experiences and contexts.

### Significant Contribution

The sixth criterion of quality research is significant contribution. Similar to a selection of a worthy topic, quality research can meaningfully contribute to the field of study in several ways, including offering theoretical, heuristic, and practical significance (Tracy, 2010). The dimensions of *applied* and *effective* (Baer et al., 1968) are related to significant contribution. First, theoretical significance is how a study can extend or build on existing disciplinary knowledge. Second, heuristic significance inspires further investigation by igniting curiosity about the study. Last, practical significance contributes to useful knowledge of the studied phenomenon and offers action. Behavior analytic research strives to contribute to improving socially significant behavior and can contribute at levels beyond the individual.

### Ethics

Ethics is the seventh criterion of quality research identified by Tracy (2010). In qualitative research, ethics go beyond procedural ethics, such as what is required by an institutional review board, and include situational considerations of each context and relationships (e.g., the consequences of the researcher’s interactions with the participants in the study). Additionally, exiting ethics focus on how research is shared and consider how readers can be warned about the ways that research analyses could be “misread, misappropriated, or misused” (Tracy, 2010, p. 848). These concepts are reflected in the Ethics Code for Behavior Analysts (Behavior Analyst Certification Board, 2020), particularly in Sect. 6: Responsibility in Research.

### Meaningful Coherence

The final criterion of Tracy’s quality research is meaningful coherence, an interconnectedness within the study. Meaningful coherence is an alignment between the study’s design, data collection, data analysis, framework, and the overall goals of the study. In essence, meaningful coherence helps the researcher achieve the stated purpose of the study while using methods that align with theories and research paradigms. This criterion is related to the *conceptually systematic* dimension of behavior analysis (Baer et al., 1968) in that both seek to align parts to a whole. Conceptually systematic dimension calls for connecting technology to relevant principle and turning a body of technology into a discipline (Baer et al., 1968). Similarly, all aspects of a study design must interconnect and align to result in meaningful coherence.

The eight “big tent” criteria offer guidance to qualitative researchers to ensure their studies possess high quality. Although qualitative researchers vary across their epistemology and research paradigms, Tracy (2010) sought to bring forth a common language and framework that applies across these differences. Any researcher can use these criteria as a tool for design and reflection of their study and its quality. Behavior analysts should also see parallels to our own foundational seven dimensions (Baer et al., 1968) that are not intended to be duplicated but are certainly related. Both have the intention of ensuring a high level of quality.

## Applying the “Big Tent” Criteria in Research

In striving to conduct rigorous qualitative research, we chose to use Tracy’s “big tent” criteria as we designed and conducted two recent qualitative studies focused on education. Although other criteria for ensuring rigor in qualitative research exist, we selected “big tent” due to their inclusivity across diverse qualitative approaches and their flexibility. We found these criteria to be helpful throughout our inquiries, allowing for a balanced approach between rigor and flexibility. Additionally, “big tent” offered a single approach to ensure rigor across diverse qualitative methods. Therefore, we wish to share two illustrations of different research methods and highlight how both studies, designed with considerations for these “big tent” criteria, improved the rigor and overall quality of the studies despite the innate differences within each research approach. By sharing these illustrations, we hope to expand the dialogue within the field of behavior analysis and extend the use of qualitative methodology beyond social validity.

These examples are not intended to suggest that “big tent” criteria described serve as the only exclusive criteria for ensuring rigor. Nor should the described research approaches themselves be considered as the only examples of rigorous qualitative research. Instead, we hope to offer two compelling examples of how researchers in the field of behavior analysis can make valuable contributions through methodologically sound and rigorous qualitative research by using a set of quality criteria.

### Example Study 1: Phenomenology

Special education administrators are frequently tasked with creating and providing professional development (PD) opportunities for educators to support their practices. Often special education teachers feel unsupported by their administrators, a perception that contributes to high rates of teacher attrition (Billingsley & Bettini, 2019). Therefore, focusing on meaningful PD is a worthy topic.

We were particularly interested in investigating how special education administrators designed this task for educators working with students with autism spectrum disorder (Layden et al., 2022, 2023). We were interested in their lived experiences: how they made meaning of their experiences, identified how and why they made decisions about providing PD, and how they viewed the implementation of those decisions. Given our focus on exploring the lived experiences of special education administrators, we determined that interpretive phenomenology best aligned with our inquiry.

Phenomenology is a qualitative method focused on understanding individual experiences and their meanings around a common phenomenon experienced by the participants (Batur & Ozcan, 2020; Creswell et al., 2007; Tavakol & Sandars, 2025). It seeks to understand the characteristics that define a particular experience or its “essence” (Tavakol & Sandars, 2025, p. 1442). Phenomenological research is grounded in two philosophical perspectives: descriptive and interpretive (Tavakol & Sandars, 2025).

Descriptive phenomenology, developed by Husserl, aims to understand the universal essences of people’s lived experiences without the researcher’s interpretation or any previous theories or frameworks (Shorey & Ng, 2022; Tavakol & Sandars, 2025). Husserl suggested that humans hold their experiences within their consciousness prereflectively (Willis et al., 2016). It is by reflecting and describing the common features of their lived experiences of the same phenomenon that researchers can discover the universal essences at the heart of the nature of the phenomenon (Shorey & Ng, 2022). Interpretative phenomenology, founded by Heidegger, shifts the researcher’s role (Tavakol & Sandars, 2025). It relies on the researcher not only to describe, but to interpret how the participants understand their experiences. Unlike Husserl, Heidegger argued that it is impossible to separate the researcher from the interpretation process and that it is the researcher’s knowledge and assumptions that guide the interpretations of the participants’ experiences (Smith & Osborn, 2015).

In our interpretive phenomenological study, the phenomenon under study referred to designing and implementing PD. Additionally, we used the implementation science model offered by Fixsen et al. (2015) as a conceptual framework for our study. A theoretical framework serves as a roadmap for a study, providing a foundation for all other aspects of the research design (Antonenko, 2015). However, this theoretical framework serves as a foundation for the a priori codes. A priori codes are codes that are developed before data analysis begins, generally using previous literature (Bingham & Witkowsky, 2021). Additional codes were allowed to emerge from our data as we engaged in initial coding. As we continued to analyze each transcript for each participant, we developed emergent themes, searched for connections across them, and then looked for patterns across the participants. This data analysis followed the steps of interpretive phenomenological analysis as described by Smith et al. (2009).

In our study of special education administrators (Layden et al., 2022, 2023), we focused on 10 participants who worked as special education administrators for at least three years in a public school setting and self-reported being in a role where their responsibilities included the development and implementation of PD opportunities for educators who supported students with ASD.

#### Findings

The data from our study were so rich, we needed to break the findings into two different manuscripts, each providing room to address the interconnected themes and offer thick description of the results, a characteristic of credibility. Between the two articles (Layden et al., 2022, 2023), we developed nine themes that included: (a) deep commitment to coaching; (b) participant-centered training; (c) systemic barriers to effective training; (d) the gap of administrator knowledge and application; (e) COVID-19 impact; (f) collaborative positioning; (g) commitment to removing barriers; (h) broken bridge between knowledge and practice; and (i) duality of systems. Each of these themes is explained in detail and supported through participants’ quotes and examples in the articles (Layden et al., 2022, 2023). From these themes, we began to understand how and why participants engaged in many of their behaviors surrounding the development and implementation of PD.

### Example Study 2: Grounded Theory

Another illustration of qualitative research is a grounded theory study, exploring the experiences of school-based behavior analysts (SBBAs) in their current professional roles (Layden et al., in press). Despite school districts hiring more behavior analysts to support students and educators, we still know very little about their roles and responsibilities and their needed supports (Layden et al., 2024). As such, improving understanding of the SBBAs’ perceptions and experiences can lead school districts to offer more effective ways of supporting them, thus maximizing their impact.

Given the limited existing research about SBBAs and a lack of an existing and related theoretical framework, we chose to design and conduct a grounded theory study to answer our research question: *How do SBBAs experience their current professional roles?* Grounded theory can be useful when little is known about a given phenomenon (Tie et al., 2019). In our study, we identified the phenomenon as the experiences of working as a full-time behavior analyst in a public school setting.

Grounded theory is a “specific methodology on how to get from systematically collecting data to producing a multivariate conceptual theory” (Glaser, 2010, p. 1). Data are systematically co-constructed by a researcher and the participants (Strauss & Corbin, 1998). The data generation and analysis occur simultaneously as researchers consistently weave the two together. Such interplay allows the researcher to refine the theory as additional data are generated, and to continue to generate data until theoretical saturation, a point where the researcher notes consistent similar patterns within the data, is reached (Cullen & Brennan, 2021; Miller, 2015; Strauss & Corbin, 1998). This interplay of interconnected data generation and data analysis is called constant comparative analysis, a cornerstone of grounded theory (Rosenbaum et al., 2016).

Within constant comparison, as data are generated, the researchers fracture them into smaller parts to capture each discrete idea (Tie et al., 2019). Next, they seek to identify patterns and relationships between these smaller parts, looking for central ideas and combining discrete ideas into concepts and categories (Strauss & Corbin, 1998). As data analysis progresses, the levels of analysis become more abstract, and researchers break down and rebuild connections within the data to build a substantive local theory (Sebastian, 2019). Strauss and Corbin (1998) offered an analogy of a puzzle to explain grounded theory. Before a puzzle can be completed, the pieces must be sorted by shape or color and connections must be looked for between them. Eventually, by connecting small pieces, parts of an image become evident, and with an ongoing trial and error process, time, and effort, a complete image of a puzzle emerges. For example, in the study of SBBAs when participants discussed advising individual teachers about supports for students with interfering behaviors, researchers created an initial label of *advising teachers*. When participants described advising school teams about student behavior, the researchers offered a label of *advising team*. As more data were generated and with constant comparison the researchers combined the labels to form the concept of *consultation*, based on the participants describing similar experiences whether they were advising individuals or teams.

Although several variations of grounded theory exist (e.g., traditional, Straussian, constructivist), this research approach rests on several core tenets that all variations have in common: coding, development of categories, constant comparison of data, theoretical sampling, theoretical saturation, theoretical integration, and the use of memos (Cullen & Brennan, 2021). When researchers follow these core tenets, they ensure abundant and complex data collection, analysis, and time in the field.

#### Findings

The findings from this study proposed a local theory with insights and a new model for how role definitions, contextual alignment, and support structures influenced the SBBAs’ perceptions of their efficacy and connected emotional responses. An improved understanding of the SBBAs’ perceptions and experiences captured by the resulting substantive local theory in this grounded theory study can lead to school districts offering more effective ways of supporting their behavior analysts, thus maximizing their impact.

### “Big Tent” Criteria Embedded in the Examples

By embedding “big tent” criteria throughout both studies, we ensured rigor in each example. Recall that the criteria included worthy topic, rich rigor, sincerity, credibility, resonance, significant contribution, ethics, and meaningful coherence.

#### Worthy Topic

Both studies investigated worthy topics with practical significance to educators. The findings from the phenomenology study contributed to an understanding of how special education administrators developed and implemented PD, which can help design learning opportunities that teachers find meaningful, thus potentially influencing teacher retention. Meanwhile, the grounded theory study led to a novel understanding of SBBAs’ experiences and their needs. Additionally, each study contributed to the paucity of research on each topic.

#### Rich Rigor

Both study designs generated abundant data, one of Tracy’s aspects of rich rigor. In the phenomenology and grounded theory studies, we conducted two rounds of semi-structured interviews with each participant. This type of interview begins with predetermined and open-ended questions but allows for follow-up questions based on what participants share (Cohen & Crabtree, 2006; Edwards & Holland, 2013). The second round of interviews provided researchers with an opportunity to ask additional questions after the initial data analysis, allowing them to explore the depth of participants’ experiences. During those interviews and accompanying data analysis, we grew confident that theoretical saturation had been reached as participants shared numerous examples of similar concepts without offering any new conceptual information.

In each study, all of the researchers involved were well-versed in both the field of ABA and education, most having practiced as behavior analysts within the public school setting and having designed and delivered PD to teachers of students with ASD. These backgrounds enabled researchers to analyze data in a manner that we believe effectively captured the nuances of the participants’ experiences, thus offering requisite validity, which further increases the rich rigor.

#### Sincerity

Throughout both studies, the research team engaged in consistent discussions about data analysis, ensuring they approached data with much thoughtfulness. For example, in the grounded theory study, the researchers continuously asked one another if their interpretations reflected the experiences of the participants of the study or the researchers’ own, given that several members of the research team had previously worked as SBBAs. Similarly, the authors of the phenomenology study had experienced working as special education administrators and planned professional development opportunities for others. In this study as well, the researcher had to remind themselves to bracket their experiences and instead focus on analyzing the data generated from the participants. In both examples, discussions about data analysis increased the reflexivity of researchers, thus improving the sincerity of the study. Additionally, both published studies discussed the researchers’ positionality.

#### Credibility

After each interview in both studies, the interviewers engaged in member checking by sending a summary to each participant to review for accuracy. Participants were asked to correct anything that they felt was inaccurate or to add anything they had not included during the interviews, offering member reflections, one means of ensuring credibility of the findings. Such summaries are essential to the credibility of research given that the participants are seen as experts in their own lived experiences and therefore their voices need to be captured as accurately as possible.

In both studies, researchers engaged in data source triangulation by interviewing 10–15 participants. Additionally, researchers used thick description, often drawing on rich quotes from different participants to capture their experiences and honor multivocality when reporting results in both articles. Additionally, although all participants in the grounded theory were employed full-time by a public school district, their roles and official job titles varied. This diversity in roles offered multivocality in different perspectives, all connected to practicing behavior analysis in the schools, thus increasing the credibility of the results.

A unique element of embedding credibility of the results into our study design in grounded theory occurred following data analysis after the second interview. Once the research team described the local theory, we conducted focus groups for the participants to provide feedback, critique, and ask questions about data analysis and the local theory. Although not all participants could attend the focus group, each SBBA had the opportunity to discuss the study’s findings individually with a researcher via a virtual meeting or provide a written response by email. All participants provided feedback on the study’s results, confirming that the findings captured their experiences and perceptions. Such member reflections increased the credibility of the study's findings.

#### Resonance

As discussed by Tracy, aesthetic merit results from the researcher reporting the results in a manner that engages a reader and tells a story. As described above, both research articles used participants’ rich quotes to capture their experiences in a way that described their experiences with aesthetic merit.

#### Significant Contribution

Deep understanding of a phenomenon under study can ignite curiosity for future research and may encourage others to pose more questions, thus making a heuristic contribution. Future research can begin to focus on components to improve not only the experience of professionals such as our participants, but of those they influence, namely the teachers working with students. Research questions related to how to remove barriers, reduce the gap between administrator knowledge and application, break down the duality of systems (between general and special education), and support SBBAs in their settings can emerge to be studied and provide additional significant practical contributions to our field. Additionally, school leaders who employ behavior analysts may be able to transfer some of the knowledge into their context and adapt their practices to support these professionals, thus increasing the study’s practical contribution.

#### Ethics

Within the field of behavior analysis, following the Ethics Code for Behavior Analysts (Behavior Analyst Certification Board, 2020) ensures that strong ethics are embedded in all our practices, including conducting research. Additionally, both studies were approved by the institutional review board. In sharing the results of both studies, the researchers took much caution to explain the qualitative research design to ensure that the findings were not misused or misunderstood.

#### Meaningful Coherence

In both studies, we ensured the alignment of inquiry, research design, and a conceptual framework offered meaningful coherence. However, distinct differences in the study design emerged on the basis of the selected research approach for each study.

In the interpretative phenomenology study, we used the implementation science model offered by Fixsen et al. (2015) as a conceptual framework for initial coding. We used a systematic data analysis process that included four steps (Wertz, 2005) and mirrored the interpretative phenomenological analysis process described by Smith et al. (2009) to analyze the interview transcriptions. First, we reviewed and re-read all the transcripts to get a sense of the whole before breaking participants’ statements into units of meaning. We determined a priori codes from the implementation science model (Fixsen et al., 2015) to use them for initial coding. During the second step, we examined each phrase in the transcripts and assigned a code to each one to begin to sort and make meaning of the data. We developed supplemental emergent codes based on the participants’ ideas when a priori codes did not capture their experiences. The third step involved reflecting on each unit of meaning to gain insight and develop emergent themes for each participant. Finally, a synthesis of the data was created to develop connections between the themes and identify overarching themes. This data analysis process mirrored interpretative phenomenological analysis as described by Smith et al. (2009). It offered meaningful coherence to the study by aligning the design with the conceptual framework.

In contrast, data generation and analysis within the grounded theory study followed the unique design of the Straussian grounded theory, which calls for researchers purposefully not to use a conceptual framework so that the local theory can be grounded in the experiences and perspectives of the participants (Sutcliffe, 2016) rather than force data to fit existing frameworks (Glaser & Strauss, 1967).

Additionally, given the tenets of grounded theory, data generation and analysis varied significantly from that in phenomenology. One of the key tenets of grounded theory is constant comparison. Within constant comparison, as data are generated, the researchers fracture them into smaller parts to capture each discrete idea (Tie et al., 2019). During this coding stage unlike in our phenomenology study, no a priori codes were used. Instead, we used in vivo codes, which use participants’ words as initial codes (Strauss & Corbin, 1998). Next, we sought patterns and relationships between these codes, looking for central ideas and combining discrete ideas into concepts and categories (Strauss & Corbin, 1998). As data analysis progressed, the levels of analysis became more abstract, and researchers broke down and rebuilt connections within the data to build a substantive local theory (Sebastian, 2019).

Although on the surface data analysis in phenomenology and grounded theory share similarities in that data are coded and connections are made within data, in practice, the two processes vary greatly. Therefore, the alignment within the research approach involves ensuring that the methodology corresponds with the research question and the purpose of our study, thus creating meaningful coherence.

## Conclusion

As demonstrated, the “big tent” criteria served as a foundational guide throughout the inquiry process and helped us ensure high quality of the research in both studies we have discussed. These criteria provide a valuable framework for navigating qualitative research, helping researchers conduct qualitative studies that are both methodologically rigorous and deeply meaningful. Although qualitative research is time-consuming, its capacity to capture the rich, multifaceted nature of human experiences is unparalleled.

We believe that researchers within the field of behavior analysis have much to gain from embracing qualitative methodologies. These types of methodologies can provide deeper insights into human behavior and are particularly well-suited for questions that SCD and other quantitative methods may not be effective in studying. Therefore, more researchers can and should consider conducting rigorous qualitative research to elevate the voices of the participants and relate the depth and complexities of their nuanced experiences. Amplifying the voices of individuals we support, serve, and work with can help improve their quality of life and enrich the existing research in our field.

It is worth acknowledging that quantitative and qualitative research are often placed in direct opposition; however, this dichotomy overlooks the potential for these methods to complement each other. We propose that they are not incompatible. The field of behavior analysis stands to benefit from leveraging the strengths of both quantitative and qualitative methodologies to gain a more comprehensive understanding of human behavior. By valuing both, we can foster a more inclusive, rigorous, and impactful body of research that can drive the field forward.

## Data Availability

There are no participants as part of this manuscript, and it is not original research.
